# New models for human disease from the International Mouse Phenotyping Consortium

**DOI:** 10.1007/s00335-019-09804-5

**Published:** 2019-05-24

**Authors:** Pilar Cacheiro, Melissa A. Haendel, Damian Smedley, the International Mouse Phenotyping Consortium and the Monarch Initiative, Terrence Meehan, Jeremy Mason, Hamed Haseli Mashhadi, Violeta Muñoz-Fuentes, Glauco Tocchini, Kent K. C. Lloyd, Colin McKerlie, Lynette Bower, Dave Clary, Lauryl M. J. Nutter, Ann M. Flenniken, Lydia Teboul, Gemma Codner, Sara Wells, Yann Herault, Tania Sorg, Laurent Vasseurm, Mohammed Selloum, Michel Roux, Hugues Jacobs, Hamid Meziane, Marie-France Champy, Ghina Bou About, Steve Murray, Elissa Chesler, Vivek Kumar, Jacqui White, Robert E. Braun, Arthur L. Beaudet, Mary E. Dickinson, Jason D. Heaney, Isabel Lorenzo, Denise G. Lanza, Corey L. Reynolds, Christopher S. Ward, Rodney C. Samaco, Surabi Veeraragavan, Chih-Wei Hsu, Audrey E. Christianson, Juan J. Gallegos, John Richard Seavitt, Angelina Gaspero, Jennie R. Green, Garza, Arturo Garza, Ritu Bohat, Radislav Sedlacek, Steve D. M. Brown

**Affiliations:** 1 William Harvey Research Institute, School of Medicine and Dentistry, Queen Mary University of London, London, UK; 2 Linus Pauling Institute and the Center for Genome Research and Biocomputing, Oregon State University, Corvallis, USA

## Abstract

The International Mouse Phenotyping Consortium (IMPC) continues to expand the catalogue of mammalian gene function by conducting genome and phenome-wide phenotyping on knockout mouse lines. The extensive and standardized phenotype screens allow the identification of new potential models for human disease through cross-species comparison by computing the similarity between the phenotypes observed in the mutant mice and the human phenotypes associated to their orthologous loci in Mendelian disease. Here, we present an update on the novel disease models available from the most recent data release (DR10.0), with 5861 mouse genes fully or partially phenotyped and a total number of 69,982 phenotype calls reported. With approximately one-third of human Mendelian genes with orthologous null mouse phenotypes described, the range of available models relevant for human diseases keeps increasing. Among the breadth of new data, we identify previously uncharacterized disease genes in the mouse and additional phenotypes for genes with existing mutant lines mimicking the associated disorder. The automated and unbiased discovery of relevant models for all types of rare diseases implemented by the IMPC constitutes a powerful tool for human genetics and precision medicine.

## The role of the IMPC in deciphering gene function and human disease

The IMPC aims to characterize the function of every protein coding gene and use the phenotypic information obtained through extensive phenotyping protocols to identify new models for human disease through cross-species (mouse-to-human) comparison. Frequent data releases are made publicly available for the research community, and the systematic comparison of phenotype abnormalities observed in the mouse with those clinical phenotypes described in humans allows for the automatic identification of suitable disease models. As the IMPC phenotyping screen expands to new gene knockout strains and novel gene–disease associations and further and improved phenotypes are reported, the potential for the IMPC to discover new models for Mendelian disease increases accordingly.

This comprehensive, standardized phenotyping screen is designed to identify and characterize phenotypic abnormalities associated to each gene knockout. The subsequent analysis provides novel insight into mammalian gene function and leads to the identification of potential genes involved in specific biological systems, e.g. auditory dysfunction (Bowl et al. 2017), abnormalities of metabolism (Rozman et al. 2018) or ophthalmic disease (Moore et al. 2018). Just as importantly, genes with pleiotropic effects across physiological systems are being described, a phenomenon which has proved to be abundant in common complex traits (Sivakumaran et al. 2011; Gratten and Visscher 2016) and of particular relevance in congenital disorders (Ittisoponpisan et al. 2017).

High-throughput viability screens are also being performed, enabling the identification of genes essential for survival in the mouse, again substantially improving our understanding of congenital diseases (Dickinson et al. 2016). A first extensive report on novel mouse models for known Mendelian disorders from the IMPC has been previously published (Meehan et al. 2017). This analysis was based on 3328 gene knockouts characterized through the IMPC pipeline (Data Release 5.0; August 2016). A total of 360 disease models were identified, revealing that approximately 20% of mouse–human orthologs associated to rare disorders showed phenotypic overlap with the human disease according to the automated PhenoDigm algorithm (Smedley et al. 2013), with this percentage increasing to 40% when embryonic and neonatal lethal phenotypes were considered.

**Fig. 1:**
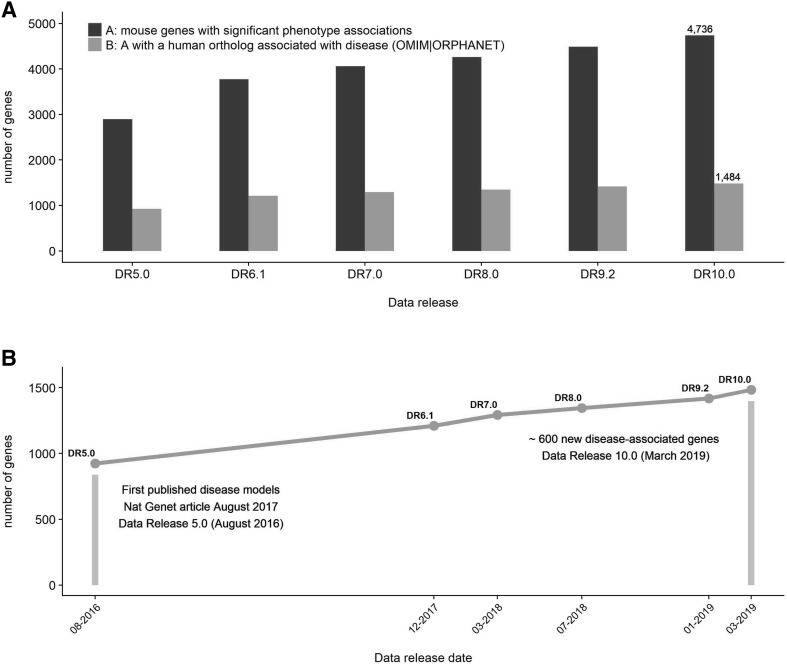
Evolution of the number of genes with null mutant mice produced and phenotyped by the IMPC over the last years. **a** Number of knockout genes with significant phenotype associations and the corresponding number of genes with human orthologs associated with disease according to OMIM or ORPHANET resources. **b** Time between data releases and number of IMPC genes with a phenotype and orthology to a known human disease gene

During the last year, frequent data releases have been made publicly available through the IMPC website, with 5861 mouse genes fully or partially phenotyped to date (DR 10.0; March 2019). A total number of 69,982 phenotype calls are reported, resulting in 4736 gene knockouts with at least one phenotypic abnormality detected through the early adult phenotyping pipeline or the viability primary (postnatal) and secondary (embryonic) screens. Once we obtain the corresponding mouse-to-human orthologs, we can observe that approximately 1/3 of those genes (1484) are associated with rare monogenic diseases as described in the OMIM (Amberger et al. 2019) and Orphanet (Rath et al. 2012) databases, a 67% increase since the first report on disease models was published (Meehan et al. 2017) and covering approximately one-third of the known Mendelian disease genes (Fig. 1).

## From mouse phenotypes to models of disease

The mouse phenotypic data collected by the IMPC are integrated with human disease resources using automated, phenotype similarity calculations. Once we have a set of significant phenotypic abnormalities associated with each gene (encoded as Mammalian Phenotype Ontology terms (MP; Smith and Eppig 2015), we can compare how similar they are with respect to the human (clinical) phenotypes, encoded by the Human Phenotype Ontology (HPO; Köhler et al. 2019), reported for the disorder associated to the human ortholog of that gene.

This phenotypic similarity is computed by several algorithms developed by the Monarch Initiative (Mungall et al. 2017). Firstly, we need to make the two ontologies comparable by correlating every MP term with the corresponding HPO term. Secondly, we compute a score which give us the similarity for each HPO–MP phenotypic match. The PhenoDigm algorithm (Smedley et al. 2013) uses all the individual scores for each HPO–MP association, taking into account the proximity of the two terms in the ontology and the frequency of the phenotype in common from the overall mouse and disease annotations. It finally produces a percentage score by comparing these results to the best possible score (a mouse model perfectly matching the disease). This score allows for the automated identification of mouse models of disease.

**Fig. 2:**
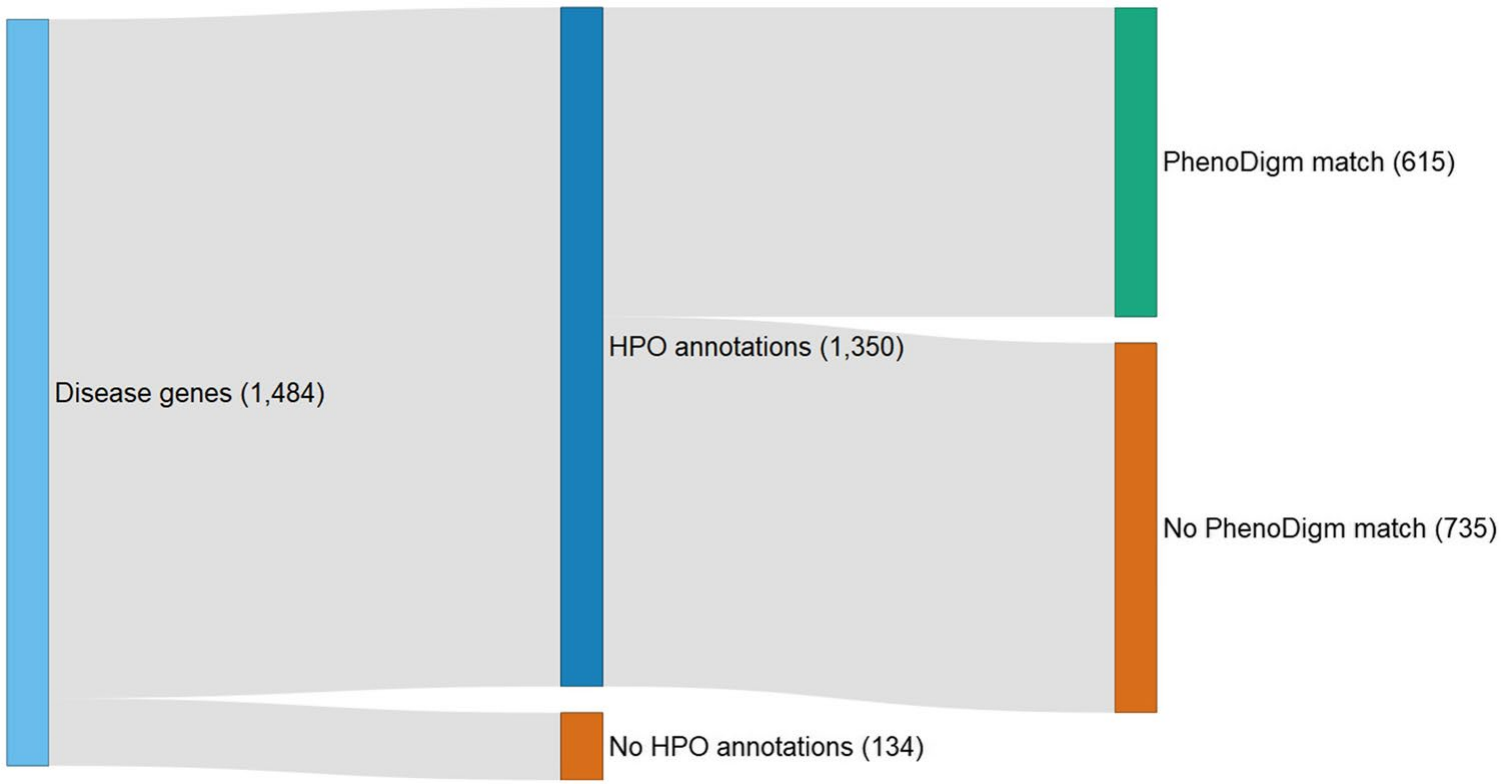
Disease-associated genes with phenotypic abnormalities described in DR10.0 and potential models for human disease. Disease genes: IMPC knockout mice with significant phenotype annotations and a human ortholog associated to disease according to OMIM or Orphanet; No HPO annotations: No HPO-encoded phenotypes available for the disorders associated to the gene; HPO annotations: HPO-encoded phenotypes available for the associated disease; No PhenoDigm match: no phenotypic similarity was found between the IMPC knockout mouse and the human clinical phenotypes; PhenoDigm match: the IMPC null mutant recapitulates at least partially the disease phenotypes

When we analyzed the data from the latest release (DR 10.0; March 2019), we identified 1484 mouse knockouts with significant phenotype annotations whose human ortholog is associated to disease. For a small percentage (134, 9.03%), there were no HPO-encoded phenotypes available for the corresponding disorder, so it was no possible to compute the phenotypic similarity and therefore their suitability as models for the disease (Fig. 2). One reason for this may be that these are very recent gene–disease associations or entries in the respective databases and the reported phenotypes have not yet been encoded as HPO terms. Manual inspection revealed, nonetheless, the mutant mouse line is able to mimic some of the disease phenotypes, e.g. the homozygous *Cfap69* IMPC line has a male infertility phenotype and the human orthologue is associated to Spermatogenic failure 24 (OMIM:617959) (Dong et al. 2018). Another example is the homozygous *Cxcr2* line with several phenotypes involving B cell and T cell disturbances and the corresponding human orthologue *CXCR2* associated to Autosomal recessive, severe congenital neutropenia due to CXCR2 deficiency (ORPHA:420699). In some other cases, we found that these are gene–phenotype relationships reported as susceptibility factors with no associated HPO phenotypes available, e.g. {Asthma, susceptibility to, 1} (OMIM:607277) and *PTGDR,* for which the IMPC mouse orthologue *Ptgdr* shows decreased basophil cell number and decreased circulating alanine transaminase level.

Considering those other genes with HPO-encoded phenotypes for the associated disorders, for 735 (49.53%) we did not obtained a PhenoDigm match, which indicates that there is no phenotype in common between the knockout mouse and the disease. This may be explained by several factors, including the following: (1) underlying genetic and physiological differences between mouse and human, e.g. those regarding gene families, which could lead to paralogous genetic redundancy in the mouse, this being the reason why the knockout is not able to recapitulate the human phenotype (Barbaric et al. 2007); (2) IMPC mouse knockouts are null mutants (loss-of-function), whereas disease-causing mutations in humans may have other effects on function (e.g. gain-of-function, dominant negative); (3) the phenotype screen is not yet completed and the relevant physiological systems still need to be evaluated; (4) algorithm limitations to capture the similarity between mammalian and human phenotypes; (5) limited coverage of the phenotypic screening pipeline for certain more specific human phenotypes (e.g. intellectual disability-related phenotypes).

For the remaining 615 genes (41.44% of the total set of disease-associated genes), the IMPC mouse mimics, at least partially, the disease phenotypes. This means an increase of 255 mutant strains regarding the number of potentially relevant disease models, and a percentage very similar to that reported in Meehan et al. (2017) (360/889; 40.49%).

## Novel mouse models for human disease

A summary of some new IMPC disease models with respect to the previous report is provided in Table 1. These examples were selected based on one of two criteria: either they are the first mouse mutant reported for that gene [i.e. there is no previous mouse model according to the Mouse Genome Informatics (MGI) resource (Smith et al. 2017)], or the IMPC knockout is able to capture new and relevant disease phenotypes.

**Table 1:** Novel disease models from the IMP**C**

Mouse gene (zygosity) Human ortholog	Disease	IMPC mouse phenotypes (MP)	Disease phenotypes (HPO)	Previous mouse mutants (MGI)	Other reported phenotypes (MP) (MGI)
*Upf3b* Hom/Hem *UPF3B*	OMIM:300676 Mental Retardation, X-Linked, Syndromic 14	Abnormal behaviour; **abnormal sternum morphology**; abnormal stomach morphology; decreased freezing behaviour; decreased leukocyte cell number; decreased lymphocyte cell number; hyperactivity, impaired contextual conditioning behaviour; impaired cued conditioning behaviour; increased fasted circulating glucose level; increased vertical activity; small kidney; small thymus	Abnormality of the musculature; Arachnodactyly; Frontal bossing; Growth abnormality; High palate; Hypoplasia of the maxilla; Intellectual disability; **Kyphosis**; Long face; Long foot; Macrocephaly; Mandibular prognathia; **Narrow chest;** Narrow face; Nasal speech; **Pectus carinatum; Pectus excavatum**; Prominent forehead; Prominent nasal bridge; **Scoliosis**; X-linked recessive inheritance	Yes	Abnormal behaviour; abnormal sleep behaviour; abnormal sleep pattern; decreased dendritic spine density; decreased frequency of paradoxical sleep; decreased prepulse inhibition; decreased slow-wave sleep duration; decreased startle reflex; impaired contextual conditioning behaviour; impaired cued conditioning behaviour; impaired neuron differentiation; increased neuronal precursor proliferation; limb grasping
*Rab3gap2* Hom *RAB3GAP2*	OMIM:212720 Martsolf Syndrome	Abnormal behaviour; **abnormal optic disk morphology;** decreased body length; **decreased bone mineral content; decreased bone mineral density**; **decreased heart rate**; decreased lean body mass; decreased total body fat amount; hyperactivity; increased circulating bilirubin level; **narrow eye opening**; **prolonged RR interval**	Abnormal toenail morphology; Autosomal recessive inheritance; Brachycephaly; Broad fingertip; Broad nasal tip; **Cardiomyopathy**; **Cataract**; **Congestive heart failure**; Cryptorchidism; Depressed nasal bridge; Downslanted palpebral fissures; Epicanthus; Feeding difficulties in infancy; High palate; Hypogonadotropic hypogonadism; Hypoplasia of the maxilla; Intellectual disability, progressive; Intellectual disability, severe; **Joint laxity**; **Lumbar hyperlordosis**; Metatarsus adductus; Microcephaly; Micrognathia; Micropenis; Misalignment of teeth; **Pectus carinatum**; **Pectus excavatum**; Posteriorly rotated ears; Prominent antitragus; Prominent nipples; Recurrent respiratory infections; Short metacarpal; Short palm; Short phalanx of finger; Short philtrum; Short stature; Short toe; Slender ulna; Talipes equinovarus; **Talipes valgus**; Tracheomalacia	No	
*Hmcn1* Hom *HMCN1*	OMIM:603075 Macular Degeneration, Age-Related, 1	Abnormal behavioural response to light; **abnormal optic disk morphology; cataract**	Autosomal dominant inheritance; **Choroidal neovascularization**; **Foveal hypopigmentation**; **Geographic atrophy**; **Macular degeneration**; **Macular drusen**; **Macular haemorrhage**; **Progressive visual loss**	No	
*Dsg2* Hom *DSG2*	OMIM:612877 Cardiomyopathy, dilated, 1BB	Abnormal coat appearance; **decreased cardiac muscle contractility; dilated heart left ventricle; increased heart weight**	**Dilated cardiomyopathy**	Yes	Abnormal cell death; decreased fibroblast proliferation; embryonic lethality at implantation, complete penetrance; embryonic lethality between implantation and somite formation, incomplete penetrance; increased susceptibility to induced colitis
*Mtnr1b* Hom *MTNR1B*	OMIM:125853 Diabetes Mellitus, Noninsulin-Dependent	**Increased fasted circulating glucose level**	Autosomal dominant inheritance; Decreased waist to hip ratio; **Insulin resistance**; Late onset; **Type II diabetes mellitus**	Yes	No abnormal phenotype detected
*Cpa1* Hom *CPA1*	ORPHA:676 Hereditary Chronic Pancreatitis	**Decreased erythrocyte cell number; decreased neutrophil cell number; increased lymphocyte cell number; increased mean corpuscular haemoglobin; increased mean corpuscular volume**	Abdominal pain; Abnormal enzyme/coenzyme activity; Diabetes mellitus; Elevated C-reactive protein level; Jaundice; **Leukocytosis**; Pancreatic calcification; Recurrent pancreatitis; **Splanchnic vein thrombosis**	Yes	No abnormal phenotype detected

Examples of new IMPC disease models with respect to the previously published report

*Hom* homozygote, *Hem* hemizygote, *Het* heterozygote

*Upf3b*: skeleton phenotypes in bold; *Rab3gap2*: eye, cardiovascular and skeleton phenotypes in bold; *Hmcn1*: eye phenotypes in bold; *Dsg2*: cardiovascular phenotypes in bold; *Mtnr1b*: endocrine/metabolism phenotypes in bold; *Cpa1*: hematopoietic/blood and blood-forming tissues phenotypes in bold

*UPF3B*, associated to an X-linked syndromic mental retardation (OMIM:300676), illustrates the added value of the IMPC for these type of disorders. A previously described mouse model shows several neurological and behavioural phenotypes. The IMPC null mutant is able to capture some of these behavioural phenotypes and, additionally, very specific skeletal abnormalities reported in patients. Similarly, *RAB3GAP2,* associated to Martsolf Syndrome (OMIM:212720), has no previous mouse mutant produced, with the novel IMPC model reflecting the pleiotropy of the gene by mimicking cardiovascular, facial/eye and skeletal phenotypes.

Other novel disease models reveal phenotypic abnormalities restricted to a particular physiological system, e.g. *HMCN1*, linked to age-related Macular Degeneration (OMIM:603075), and with several vision/eye phenotype associations found in the mouse mutant, including cataract, abnormal optic disk morphology as well as an abnormal behavioural response to light; or *DSG2,* associated to Cardiomyopathy, dilated, 1BB (OMIM:612877) for which the ortholog homozygous knockout mouse shows several cardiovascular phenotypes: decreased cardiac muscle contractility, dilated heart left ventricle and increased heart weight. Another example is *MTNR1B*, related to noninsulin-dependent Diabetes mellitus (OMIM:125853), and the corresponding IMPC mouse line showing increased fasted circulating glucose levels. Finally, an inherited form of pancreatitis, Hereditary Chronic Pancreatitis (ORPHA:676), for which mutations in *CPA1* have been found to be associated with, provides another example of a prospective disease model, with no previous knockout mouse able to capture the clinical phenotypes and the IMPC homozygous mice displaying several phenotypes related to pancreatic function, e.g. abnormal neutrophil and lymphocyte cell numbers.

## Ongoing and future work

Novel gene–phenotype associations covering diverse biological systems will continue to be added to the IMPC catalogue to complete an encyclopaedia of mammalian gene function. This comprises both the completion of partially phenotyped lines and the addition of new null mutant mouse strains, providing novel and better characterized mouse models. The analysis of their phenotypes will enhance the collection of those models of particular significance for human disease studies.

Several undergoing projects aim to improve the identification of relevant mouse models. An embryo imaging automated analysis pipeline is being developed, where high-resolution 3D imaging is used to quantify aberrant morphology that could not be determined by gross inspection (Brown et al. 2018). This will be crucial for the automatic detection of embryo abnormalities critical for congenital anomalies and developmental disorders. Moreover, additional improvements in the phenotyping screening protocols are being made. Such advances include the implementation of a late-onset systemic phenotyping (ageing) pipeline, with the potential of revealing phenotypes modelling age-related disease, and also the identification of human phenotype areas less covered by the current mouse phenotyping screenings or those others which might be more challenging to implement.

To keep the pace with the rapid generation of data from the IMPC production and phenotyping centres, the Mouse Phenotyping Informatics Infrastructure (MPI2) (Ring et al. 2015) is performing upgrades to the software and methods used for the automated statistical analysis of phenotype data. Thus, a new window approach is currently being applied to assess the significance of the phenotypic abnormalities observed in the mutant mice. A thorough revision and improvement of the phenotype matching algorithms used for the identification of relevant disease models is also underway. This will allow, for instance, to take full advantage of the standardized phenotyping, including accounting for the absence of a given phenotype (negative phenotype).

In summary, the comprehensive phenotype screen performed by the IMPC, covering the full range of physiological systems, is not focused in any particular disease area. This, together with the interspecies comparison of phenotypes currently implemented, allows the automated, unbiased identification of models for all types of human disease. The increasing number of knockout mouse lines available, covering up to one-third of known human Mendelian disease genes, makes the IMPC catalogue a critical resource for the human genetics and precision medicine community.

## Methods

### IMPC mouse phenotypes

All the significant phenotype associations from the latest and previous data releases are publicly available from the IMPC portal.

Files: ALL_genotype_phenotype.csv.gz [Downloaded 02/05/2019]

Source: ftp://ftp.ebi.ac.uk/pub/databases/impc/

### Gene–disease associations

The human genes associated with Mendelian disease were obtained from OMIM (Amberger et al. 2019) and Orphanet (Rath et al. 2012) databases.

#### OMIM

Files: mim2gene.txt.gz; morbidmap.txt [Downloaded 21/03/2019]

Source: https://www.omim.org/downloads/

#### Orphanet

File: en_product6.xml [Downloaded 21/03/2019]

Source: http://www.orphadata.org/cgi-bin/index.php

### Human phenotypes

The human clinical phenotypes—encoded as Human Phenotype Ontology (HPO) annotations reported for these disorders, were extracted from the HPO portal (Köhler et al. 2019).

File: phenotype_annotation.tab.gz [Downloaded 21/03/2019]

Source: http://compbio.charite.de/jenkins/job/hpo.annotations/

### Mouse–human orthologues

The mouse–human orthologues were identified from Ensembl through BioMart (Hunt et al. 2018) [Ensembl95, Downloaded 21/03/2019].

### MGI mouse phenotypes

Previously reported mouse phenotypes were obtained from the MGI resource (Smith et al. 2017)

Files: MGI_PhenoGenoMP.rpt.gz; MGI_GenePheno.rpt [Downloaded 21/03/2019]

Source: http://www.informatics.jax.org/downloads/reports/index.html

### Phenotypic similarity

The PhenoDigm algorithm (Smedley et al. 2013) computes individual scores for each HPO–MP phenotypic match, based on the proximity of the two terms in the overall cross-species ontology (Jaccard index; simJ) and the observed frequency of the phenotype in common from the entire set of disease and mouse annotations (Information Content; IC). The geometric mean of the IC and simJ measures was used to generate the HPO–MP pairwise score. The overall score, which is a percentage-based score, is the result of comparing the best and mean scores for all the pairwise HPO–MP comparisons relative to the maximum possible scores for a mouse model perfectly mimicking the disease phenotypes. The disease models as described in this paper: PhenoDigm percentage score greater than 0. No PhenoDigm match: PhenoDigm percentage score equal to 0, i.e. no single HPO–MP match.

### Software

Statistics and figures were generated using R 3.5.1 (R Core Team 2018) and the following packages: ggplot2 (Wickham 2016), cowplot (Wilke 2019) and networkD3 (Allaire et al. 2017).
